# Differential frequency of NKG2C/*KLRC2* deletion in distinct African populations and susceptibility to Trachoma: a new method for imputation of *KLRC2* genotypes from SNP genotyping data

**DOI:** 10.1007/s00439-016-1694-2

**Published:** 2016-06-16

**Authors:** Adriana Goncalves, Pateh Makalo, Hassan Joof, Sarah Burr, Athumani Ramadhani, Patrick Massae, Aiweda Malisa, Tara Mtuy, Tamsyn Derrick, Anna R. Last, Meno Nabicassa, Eunice Cassama, Joanna Houghton, Christine D. Palmer, Harry Pickering, Matthew J. Burton, David C. W. Mabey, Robin L. Bailey, Martin R. Goodier, Martin J. Holland, Chrissy h. Roberts

**Affiliations:** Department of Clinical Research, Faculty of Infectious and Tropical Diseases, London School of Hygiene and Tropical Medicine, London, UK; Disease Control and Elimination Theme, Medical Research Council Unit, Fajara, The Gambia; Kilimanjaro Christian Medical Centre, Moshi, Tanzania; International Centre for Eye Health, London School of Hygiene and Tropical Medicine, London, UK; Programa Nacional de Saude de Visao, Ministerio de Saude Publica, Bissau, Guinea-Bissau; Department of Immunology and Infectious Disease, Faculty of Infectious and Tropical Diseases, London School of Hygiene and Tropical Medicine, London, UK

## Abstract

NKG2C is an activating receptor that is preferentially expressed on natural killer (NK) cells. The gene encoding NKG2C (killer cell lectin-like receptor C2, *KLRC2*) is present at different copy numbers in the genomes of different individuals. Deletion at the NKG2C locus was investigated in a case–control study of 1522 individuals indigenous to East- and West-Africa and the association with the ocular *Chlamydiatrachomatis* infection and its sequelae was explored. The frequency of homozygous *KLRC2* deletion was 13.7 % in Gambians and 4.7 % in Tanzanians. A significantly higher frequency of the deletion allele was found in West-Africans from the Gambia and Guinea-Bissau (36.2 % *p* = 2.105 × 10^−8^, 26.8 % *p* = 0.050; respectively) in comparison to East-African Tanzanians where the frequency of the deletion is comparable to other human populations (20.9 %). We found no evidence for an association between the numbers of *KLRC2* gene copies and the clinical manifestations of trachoma (follicular trachoma or conjunctival scarring). A new method for imputation of *KLRC2* genotypes from single nucleotide polymorphism (SNP) data in 2621 individuals from the Gambia further confirmed these results. Our data suggest that NKG2C does not play a major role in trachomatous disease. We found that the deletion allele is present at different frequencies in different populations but the reason behind these differences is currently not understood. The new method offers the potential to use SNP arrays from genome wide association studies to study the frequency of *KLRC2* deletion in other populations and its association with other diseases.

**Electronic supplementary material:**

The online version of this article (doi:10.1007/s00439-016-1694-2) contains supplementary material, which is available to authorized users.

## Introduction

Natural killer (NK) cells play a crucial role in innate and adaptive immune responses against virally infected and tumorigenic cells as well as in reproduction in placental mammals (Cerwenka and Lanier 2001; Moffett and Loke 2006). NK cell functions range from cytotoxic activity to the production of cytokines such as TNF-α and IFN-γ. This variety of functions is controlled by the integrated action of activating and inhibitory receptors at the cell surface (Lanier 2005; Long et al. 2013). NK cell receptors include the killer-cell immunoglobulin-like receptors (KIRs) and the C-type lectin-like family of receptors. A high proportion of the NK cell repertoire expresses inhibitory receptors such as the C-type lectin-like NKG2A/CD94 dimers and inhibitory KIRs that bind to HLA-E and HLA class I ligands, respectively (Braud et al. 1998; Parham 2005). The recognition of HLA ligands by inhibitory receptors on NK cells restricts NK cell activation and targeted cytotoxicity. In the context of viral infection or malignant cell transformation, the balance between inhibitory and activating signals is shifted and cells with down-regulated levels of HLA class I and HLA-E alleles become targets of activated NK cell subtypes (Lodoen and Lanier 2005; Wieten et al. 2014).

NKG2C, the activating counterpart of NKG2A, is expressed by a different subtype of peripheral blood NK cells that also recognise non-classical HLA-E. Contrary to NKG2A that delivers inhibitory signals through its cytoplasmic ITIM motif; NKG2C/CD94 heterodimers associate with the ITAM motif of the adaptor protein DAP12 to activate NK cells (Borrego et al. 1998; Braud et al. 1998; Lee et al. 1998). A unique population of mature CD57^+^NKG2A^−^NKG2C^+^ NK cells undergoes selective expansion in response to infection with human cytomegalovirus (HCMV) (Della Chiesa et al. 2012; Foley et al. 2012a, b; Guma et al. 2004, 2006; Lopez-Verges et al. 2011) and other viral infections (Hantavirus, Chikungunya, HIV and Hepatitis B) (Beziat et al. 2012; Bjorkstrom et al. 2011; Brunetta et al. 2010; Petitdemange et al. 2011). Expansion of NKG2C positive cells was also reported for non-communicable diseases, including Stevens–Johnson syndrome and psoriasis (Batista et al. 2013; Morel et al. 2010). Importantly, the expansion of NKG2C positive NK cells in response to HIV and hepatitis B infections was restricted to HCMV seropositive patients (Beziat et al. 2012; Brunetta et al. 2010).

The NKG2C receptor is encoded by the *KLRC2* gene, which is located in the NK complex (NKC) on chromosome 12p13. Heterozygous and homozygous *KLRC2* gene deletion is present in different populations in a significant proportion of individuals (Hikami et al. 2003; Li et al. 2015; Miyashita et al. 2004; Moraru et al. 2012b; Rangel-Ramirez et al. 2014; Thomas et al. 2012). Deletion at the NKG2C locus has been shown to lead to an increased risk of HIV infection and faster disease progression (Thomas et al. 2012). An association between *KLRC2* deletion and psoriasis has also been reported (Zeng et al. 2013). No apparent association has been found with other viral infections (Moraru et al. 2012b; Rangel-Ramirez et al. 2014) or with other diseases such as rheumatoid arthritis, systemic lupus erythematosus and nasopharyngeal carcinoma (Li et al. 2015; Miyashita et al. 2004). Recently, *KLRC2* deletion was shown to be associated with a reduction in the proportion of mature NK cells expressing CD94 and CD57. Furthermore, children (but not adults) bearing the *KLRC2* deletion had significantly higher levels of anti-HCMV IgG antibodies, suggesting that NKG2C plays a role in the control of HCMV infection early in childhood (Goodier et al. 2014). Interestingly, the association between *KLRC2*/NKG2C deletion and intracellular bacterial infections has not been described so far.

Several pieces of evidence suggest that NK cells play a role in the resolution of infection with *Chlamydia trachomatis* (Ct), a gram-negative obligate intracellular bacterium. Repeated ocular infection with *Chlamydia trachomatis* serovars A, B and C causes trachoma, a blinding disease characterised by chronic conjunctival inflammation in childhood that develops into conjunctival scarring and blindness later in life (Hu et al. 2010). The mechanistic relationship between chronic inflammation and conjunctival scarring is not completely understood. It has been suggested that the scarring complications of trachoma occur as a result of the burden of infection and the host immune response itself (Derrick et al. 2015). The resolution of Ct infection requires an IFN-γ dependent Th1 mediated immune response (Derrick et al. 2015; Holland et al. 1996). Ct infection was shown to activate IFN-γ production by human NK cells along with activation of IFN-γ inducing cytokines (IL-18, IL-12) by epithelial and dendritic cells (Hook et al. 2004, 2005). NK cell-mediated cytotoxicity of epithelial cells has also been described (Hook et al. 2004). In vivo studies in mice have further demonstrated that NK cells are the source of early IFN-γ production in response to genital chlamydial infection (Tseng and Rank 1998). NK cells were also shown to modulate Th1/Th17 responses induced by lung dendritic cells upon *Chlamydia muridarum* infection (Shekhar et al. 2015). In humans living in trachoma endemic regions, NK cells have further been identified as the primary source of early IFN-γ production, a response that increases with age (Gall et al. 2011). Conjunctival gene expression studies have identified NK cell activation and cytotoxicity pathways associated with active disease (Natividad et al. 2010). Of particular note is the up-regulation of genes required for NK cell responses such as different NK cell receptors (KIRs and NCR3), perforin (PRF1) and granzyme B (GZMB). A recent study demonstrated that the odds of developing scarring trachoma increase with increasing number of genome copies of HLA-C2, which is further amplified by heterozygosity at KIR2DL2/KIR2DL3 (Roberts et al. 2014). Interestingly, in NKG2C positive NK cells there is a predominance of inhibitory KIRs that recognise HLA-C ligands (Beziat et al. 2012, 2013; Djaoud et al. 2013; Foley et al. 2012b; Horowitz et al. 2013). More recently, Hu et al. have shown evidence for NK cell infiltration in the conjunctiva of patients with scarring trachoma (Hu et al. 2016). In line with the available evidence, we hypothesised that NK cells and signalling through NK cell receptors such as KIRs and NKG2C could be important risk factors for trachoma.

The frequency of the *KLRC2* deletion has been investigated in Mexican mestizos, Dutch, German, Spanish, Japanese and Chinese populations (Li et al. 2015; Miyashita et al. 2004; Moraru et al. 2012b; Rangel-Ramirez et al. 2014; Thomas et al. 2012). With the exception of the Mexican mestizo population for which the *KLRC2* deletion allele was found at a frequency of approximately 10.3 %, the frequency of the deletion haplotype seems to be comparable among the other populations studied (17.5–21.9 %). We have previously reported that in a limited number of Gambians from a single district, the frequency of NKG2C deletion was 29.3 % (Goodier et al. 2014). The reason behind the maintenance of NKG2C deletion in different populations is currently not understood and studies looking at the frequency of NKG2C deletion in different African populations are lacking.

In this study, we compare and quantify the frequency of *KLRC2*/NKG2C deletion in different African populations (East- and West-Africa) where trachoma is or has been endemic at the time of sample collection. We are the first to investigate the association between NKG2C/*KLRC2* deletion and the different stages of trachomatous disease; trachomatous inflammation-follicular (TF) a reversible clinical sign of active disease in children and trachomatous scarring (TS), believed to be irreversible, that develops over time. We also describe a new method for *KLRC2*/NKG2C genotype imputation from single nucleotide polymorphism (SNP) genotyping data.

## Methodology

### Ethics statement

The study was conducted in accordance with the Declaration of Helsinki. Samples included in the study were obtained from anonymous archived genomic DNA stocks. Permission for collection of samples/specimens and genotyping was granted by the relevant local and national ethics committees of the London School of Hygiene and Tropical Medicine, The Gambian Government/Medical Research Council Unit, the Comité Nacional de Ética em Saúde of Guinea Bissau, Kilimanjaro Christian Medical College Ethics Committee and the National Institute for Medical Research Ethics Committee in Tanzania. Written informed consent prior to a participant’s enrolment was obtained from all adult participants and from a parent or a guardian for participants aged under 18 years.

### Study populations

Participants were recruited from multiple rural regions in the Gambia, from Maasai communities in Kilimanjaro Region, Tanzania and from seven islands of the Bijagos Archipelago in Guinea-Bissau (Derrick et al. 2016a, b; Roberts et al. 2015). Genotyping was performed using buccal swab isolated genomic DNA from a subset of participants from these studies selected at random: a total of 226 children (1–13 years old) with or without trachomatous inflammation-follicular (TF) and 1296 individuals (0–103 years old) with or without trachomatous scarring (TS). In detail, a total of 62 children from the Gambia, 164 children from Guinea-Bissau, 509 Tanzanian Maasai adults and 787 Gambians (out of which 174 were children ≤13 years old) were genotyped by sequence-specific primer-PCR (SSP-PCR). Blood was further collected from a sample of 76 Gambian adults and peripheral blood mononuclear cells (PBMCs) were isolated by density gradient centrifugation for flow cytometric analysis. Clinical grades of trachoma were obtained using the WHO simplified trachoma grading system (WHO simplified trachoma grading system 2004) and the WHO 1981 FPC trachoma grading system (Dawson 1981). TF cases were identified as individuals presenting with follicular scores of “F2” or “F3” using the FPC system; whereas individuals with no clinical signs of follicles (F0), papillary hypertrophy (P0) or conjunctival scarring (C0) were identified as controls. TS cases were identified as individuals presenting conjunctival scarring in either eye according to the WHO simplified system.

### *KLRC2*/NKG2C genotyping

*KLRC2* genotypes were determined by touchdown PCR using the Phusion High Fidelity PCR kit (New England Biolabs) according to previously described methods (Goodier et al. 2014). Primer sequences were derived from the literature (Miyashita et al. 2004; Moraru et al. 2012a) with minor modifications. The primers for detection of the wild-type allele generate a 200-bp product (Forward primer: 5′-AGTGTGGATCTTCAATGATA-3′; Reverse primer: 5′-TTTAGTAATTGTGTGCATCCT-3′). The second primer pair detects the deletion allele yielding a 411-bp product (Forward primer: 5′ACTCGGATTTCTATTTGATGC3′; Reverse primer: 5′ACAAGTGATGTATAAGAAAAAG3′. When the *KLRC2* gene is present, the deletion primer pair (411 bp) fails to generate a product and only the 211-bp product within the *KLRC2* gene is detected. In the absence of the *KLRC2* gene, the distance between the deletion primers is decreased and a 411-bp product can then effectively be detected as a result of the deletion. Using this method, individuals expressing two copies of *KLRC2* (wt/wt), one copy of *KLRC2* (wt/del) or homozygous for the 16-Kb deletion (del/del) were identified. Touchdown PCR was carried out as previously described (Goodier et al. 2014). Specifically, initial denaturation was performed at 95 °C for 3 min, followed by 10 cycles of denaturation at 94 °C for 30 s, annealing for 30 s from 65 to 55 °C (65 °C on the first cycle and minus one degree per cycle) and extension at 72 °C for 30 s, followed by 26 cycles of denaturation at 94 °C for 30 s, annealing at 55 °C for 30 s and extension at 72 °C for 30 s. PCR products were separated and identified using agarose gel electrophoresis. The primers and genotyping method were optimised and validated against a panel of reference cells lines (Moraru et al. 2012a). Genotypes were assigned by blinded visual inspection of the gels in batches of up to 95 samples per assay. Unsuccessful genotyping results were not included in the analysis.

### Flow cytometry

Genotyping results were further confirmed by flow cytometry analysis of NKG2C protein expression at the surface of peripheral blood CD3 negative and CD56 positive NK cells in a subgroup of individuals from the study (*N* = 76). PBMCs were incubated with the following monoclonal antibodies: CD56 PE Cy7; CD3 V500 (BD Biosciences, Oxford, UK) and NKG2C PE (R&D Systems, Abingdon, UK) as previously described (Goodier et al. 2014). Cells were acquired on an LSRII flow cytometer using FacsDiva software (BD biosciences) and data were analysed using FlowJo (Treestar Inc., Ashland, OR, USA).

### Statistical analysis

Genetic associations were investigated, analysed and reported according to the Strengthening the Reporting of Genetic Association studies (STREGA) recommendations (Little et al. 2009). The genotyping data were analysed using R (https://www.r-project.org/) and STATA version 14.1 (StataCorp LP, College Station, TX, USA). Genotype frequencies were tested for deviation from Hardy–Weinberg equilibrium prior to the association analysis. A multivariable logistic regression was performed to investigate the association between *KLRC2*/NKG2C genotype and trachomatous disease (either TF for active disease in childhood or TS for conjunctival scarring). The association between genotype and TF was measured in West-African children from the Gambia and Guinea-Bissau. The association between genotype and TS was measured in West-African Gambians (multiple ethnicities) and East-African Tanzanian Maasai. Genotype was used as the explanatory variable and is divided in three levels (wt/wt, wt/del, del/del). Age and gender were included a priori in the model as potential confounders. Confounding by ethnic origin was controlled for in the Gambian population. Tabulation of the different variables and cross-tabulation with the outcome was used to access the distribution of the study sample and identify missing data. Missing data (≥5 %) was accounted for during the statistical analysis.

Genotype data from this study and previous published research (Li et al. 2015; Miyashita et al. 2004; Moraru et al. 2012b; Rangel-Ramirez et al. 2014; Thomas et al. 2012) were used to determine the association between origin of study population and the frequency of the deletion allele in healthy controls. Origin of population was grouped in accordance to the 1000 genomes project (http://www.1000genomes.org) into: Tanzanians (Maasai), West-Africans (Gambians and Bissau-Guineans), Europeans (Germans, Dutch and Spanish), East-Asians (Chinese and Japanese) and Mexican Mestizos. The association was determined using a logistic regression model in which the outcome was each individual allele (0/1 for wild-type and deletion alleles, respectively) and the explanatory variable was the origin of the population. Statistical hypotheses were evaluated using the Wald-test and the likelihood ratio test (LRT).

### Imputation of *KLRC2*/NKG2C genotypes from SNP genotyping data

*KLRC2* deletion variant genotypes were imputed from commercial single nucleotide polymorphism (SNP) genotyping arrays in 1090 TS cases and 1531 controls from the Gambia. All specimens had previously been directly genotyped at 1,467,876 SNPs using HumanOmni2.5-8v1_A arrays (Illumina Inc, San Diego, CA. USA) (Roberts et al. 2015). Imputation was used to increase the number of directly or indirectly genotyped SNPs to 11,851,747 SNPs, including 2,960,018 on chromosome 12. Genotyping, imputation and quality control of this data are described in a previous publication (Roberts et al. 2015). Of the 2621 specimens included in this analysis, 145 cases and 164 controls (*n* = 309) were also genotyped for the *KLRC2* deletion variant by SSP-PCR. For the 309 specimens that were included in both studies, we merged the *KLRC2* genotyping data with chromosome 12 SNP data using R and PLINK. Pairwise Linkage Disequilibrium (LD) was estimated between the *KLRC2* deletion polymorphism and each SNP in chromosome 12. All SNPs in LD with the deletion variant with LD R2 >0.2 were initially selected as candidate markers for use in a classification model. Random Forest, implemented through the Ranger package for R (Wright and Ziegler 2015), was used to estimate the relative importance of SNPs in determining the *KLRC2* genotype. The predictions of the machine-learning model (using all SNPs with R2 >0.2) were compared to the true genotypes and Cohen’s Kappa was used to determine the extent of agreement between the predicted and genotyped data. We aimed to minimise the number of SNPs required for accurate classification whilst not impacting significantly on classification accuracy. The classification model was run again using only the top 1, 2, 3 or 4 SNPs according to the estimates of variable importance. Cohen’s kappa was used, along with the number of misclassifications observed in cross-tabulation, to identify the best 1–4 SNP model that could be used to impute the *KLRC2* genotype. The model with the fewest misclassifications and highest unbiased Kappa estimate was selected as the final classifier.

Finally, we selected all specimens from the Gambian case control sample in which there were no missing data for the SNPs selected as the components of the best Random Forest model. We then applied the model to the imputation of the NKG2C genotype in 1010 cases of TS and 1419 controls. The imputed genotypes were tested for Hardy–Weinberg equilibrium using a likelihood-ratio test. Test of association between imputed *KLRC2* genotype and TS phenotype was performed using logistic regression, including gender and age as covariates.

## Results

### Validation of the NKG2C/KLRC2 genotyping method

NKG2C/*KLRC2* genotyping methods initially developed by Myashita et al. (2004) were further optimised to specifically recognise the *KLRC2* gene and used to characterise a panel of reference cell lines (Moraru et al. 2012a). The primers and genotyping method used in this study were modified from those published by Moraru et al. and have been published elsewhere (Goodier et al. 2014). In order to validate our approach and rule out the possibility of cross-amplification of highly homologous genes (e.g. the gene encoding the NKG2A receptor), we tested our primers on a set of previously published reference cell lines (Moraru et al. 2012a). Our method accurately determined the genotypes of all 14 cell lines tested (Supplementary Figure 1A). Furthermore, flow cytometry analysis in a sample of 76 individuals confirmed that absence of the *KLRC2* gene translates into lack of NKG2C expression on the surface of NK cells in all 13 individuals homozygous for the *KLRC2* deletion. In line with previously published observations (Muntasell et al. 2013; Noyola et al. 2012; Thomas et al. 2012), we found a significant correlation between the mean fluorescence intensity (MFI) of the NKG2C staining and increasing number of *KLRC2* gene copies (Supplementary Figure 1B, C). Genotyping was successful in 1522/1561 (97.5 %). Genotyping failure occurred in 15 Gambian and 24 Tanzanian specimens. The number of failures was comparable between cases and controls (not shown). All 39 individuals with unsuccessful genotyping results were excluded from the study.

### Association between *KLRC2* genotype and Trachomatous disease

A total of 1522 individuals were genotyped: 1013 from West-Africa (Gambia and Guinea-Bissau) and 509 from the Maasai community in Tanzania. The association between *KLRC2*/NKG2C deletion and TS was investigated in 787 Gambians [314 Mandinka (39.9 %); 236 Jola (30.0 %) and 237 (30.1 %) from other ethnicities] and 509 Maasai Tanzanians (Table 1). Sample size was estimated to achieve 80 % power to detect a 1.5 effect size at the 0.05 significance level in an additive genetic model. Furthermore, a total of 226 children (62 Gambians and 164 Bijagos-islanders) with and without TF were genotyped to determine the association between *KLRC2* deletion and active disease. The distribution of *KLRC2* genotypes in all three populations (Table 1) was compatible with Hardy–Weinberg equilibrium (*p* > 0.05). The association between *KLRC2* genotypes and clinical signs of trachoma was investigated by multivariate logistic regression including age and gender in the model. As expected, there was strong evidence (*p* < 0.001) that the odds of TS increase with increasing age in both populations studied (Table 2). After adjusting for age and genotype, we found evidence (*p* = 0.02) that Tanzanian females have higher relative odds of TS as compared to males (OR 1.68 95 % CI: 1.08–2.62). There was no apparent association between *KLRC2* genotype and TS in either West-African Gambians (*p* = 0.909) or East-African Tanzanian Maasai (*p* = 0.417) (Table 2). There was also no evidence of an association (*p* = 0.852) between *KLRC2* genotype and TS in West-African Gambians after adjusting for ethnic origin (Supplementary Table 1). Evidence for an association between KLRC genotype and active disease (TF) was also not found in West-African children (*p* = 0.617) (Supplementary Table 2). Overall, the data suggest that there is no association between the number of NKG2C/*KLRC2* copies and the clinical manifestations of ocular *C. trachomatis* infection in different human populations with endemic trachoma.

**Table 1:** Demographic characteristics and *KLRC2* genotypes in the different study populations

West-Africans (The Gambia)				East-Africans (Tanzania)				West-African Children (The Gambia, Guinea-Bissau)			
	TS cases (*N* = 474)	Controls (*N* = 313)	Total (*N* = 787)		TS cases (*N* = 265)	Controls (*N* = 244)	Total *(N* = 509)		TF cases (*N* = 110)	Controls (*N* = 113)	Total^b^ (*N* = 226)
Age (years)				Age (years)				Age (years)			
Mean	44.6	36.5	41.4	Mean	57.1	44.7	51.2	Mean	4.6	4.8	4.7
Range	0.5–103	0–98	0–103	Range	30–95	30–81	30–95	Range	1–13	1–13	1–13
Gender^a^				Gender				Gender			
Female	313/474 (66.0 %)	212/313 (67.7 %)	525/787 (66.7 %)	Female	188/265 (70.9 %)	174/244 (71.3 %)	362/509 (71.1 %)	Female	59/110 (53.6 %)	60/113 (53.1 %)	120/226 (53.1 %)
Male	161/474 (34.0 %)	100/313 (32.0 %)	261/787 (33.2 %)	Male	77/265 (29.1 %)	70/244 (28.7 %)	147/509 (28.9 %)	Male	51/110 (46.4 %)	53/113 (46.9 %)	106/226 (46.9 %)
Ethnicity				Ethnicity				Country			
Mandinka	200/474 (42.2 %)	114/313 (36.4 %)	314/787 (39.9 %)	Maasai	509/509 (100 %)			The Gambia	30/110 (27.3 %)	32/113 (28.3 %)	62/226 (27.4 %)
Jola	128/474 (27.0 %)	108/313 (34.5 %)	236/787 (30.0 %)					Guinea-Bissau (Bijagos)	80/110 (72.7 %)	81/113 (71.7 %)	164/226 (72.6 %)
Others	146/474 (30.8 %)	91/313 (29.1 %)	237/787 (30.1 %)								
*KLRC2* genotype				*KLRC2* genotype				*KLRC2* genotype			
wt/wt	189/474 (39.9 %)	127/313 (40.6 %)	316/787 (40.2 %)	wt/wt	174/265 (65.7 %)	155/244 (63.5 %)	329/509 (64.6 %)	wt/wt	57/110 (51.8 %)	51/113 (45.1 %)	110/226 (48.7 %)
wt/del	218/474 (46.0 %)	145/313 (46.3 %)	363/787 (46.1 %)	wt/del	80/265 (30.2 %)	76/244 (31.2 %)	156/509 (30.7 %)	wt/del	46/110 (41.8 %)	54/113 (47.8 %)	100/226 (44.2 %)
del/del	67/474 (14.1 %)	41/313 (13.1 %)	108/787 (13.7%)	del/del	11/265 (4.1 %)	13/244 (5.3 %)	24/509 (4.7 %)	del/del	7/110 (6.4 %)	8/113 (7.1 %)	16/226 (7.1%)

West-Africans (Gambians), East-Africans (Tanzanians Maasai), West-African children (Gambians *N* = 62, Bissau-Guineans *N* = 164)

*TS* trachomatous scarring, *TF* trachomatous inflammation-follicular

^a^Data missing for gender of one participant (0.1 %)

^b^Data missing for phenotype of three participants (1.3 %)

**Table 2:** Adjusted odds ratios (age and gender) for the association between *KLRC2* genotypes and trachomatous scarring (TS); estimated by logistic regression

	West-Africans (*N* = 787)				East-Africans (*N* = 509)			
	OR	95 % CI	*p* value*		OR	95 % CI	*p* value*	
Genotype								
wt/wt	1	–	–	0.908	1	–	–	0.417
wt/del	1.03	0.75–1.41	0.843		0.81	0.53–1.24	0.329	
del/del	1.11	0.70–1.75	0.663		0.62	0.25–1.55	0.307	
Age	1.02	1.01–1.02	8.325 × 10^−7^		1.07	1.05–1.08	4.466 × 10^−18^	
Gender	0.80	0.58–1.10	0.164		1.68	1.08–2.62	0.021	

West-Africans (Gambians); East-Africans (Tanzanians Maasai)

*OR* odds ratio (adjusted for all other variables in the table), *CI* confidence interval

* Wald test (left), Likelihood Ratio Test (right)

### Lack of association with scarring trachoma in Gambian adults confirmed by imputed *KLRC2* genotypes from SNP genotyping data

**Fig. 1:**
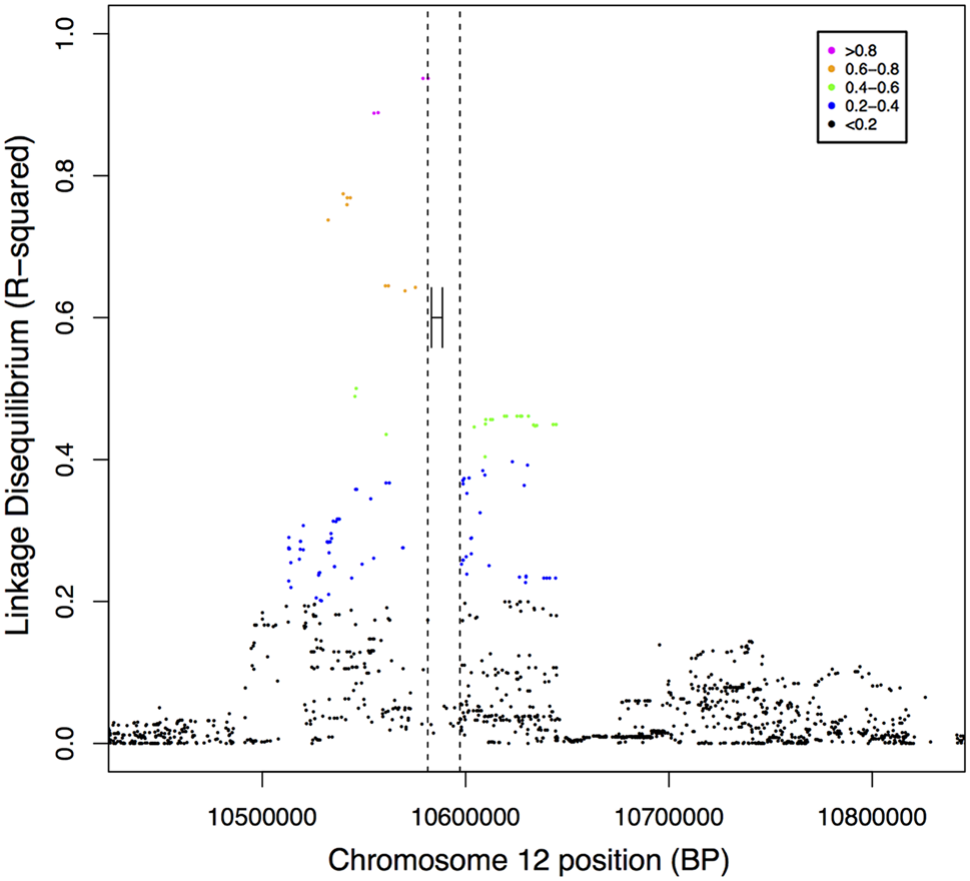
Imputation of *KLRC2* genotypes from SNPs. Patterns of LD with the deletion variant in the training set. *Dashed line* shows the limits of the deletion. *Closed lines* show the location of the NKG2C gene in wild-type chromosomes. 111 SNPs were in LD with the deletion with *R* ^2^ >0.2: 0.2–0.4 (*blue*), 0.4–0.6 (*green*), 0.6–0.8 (*amber*), >0.8 (*pink*). Nucleotide position based on URCh37.p13 Annotation release 105 (colour figure online)

**Fig. 2:**
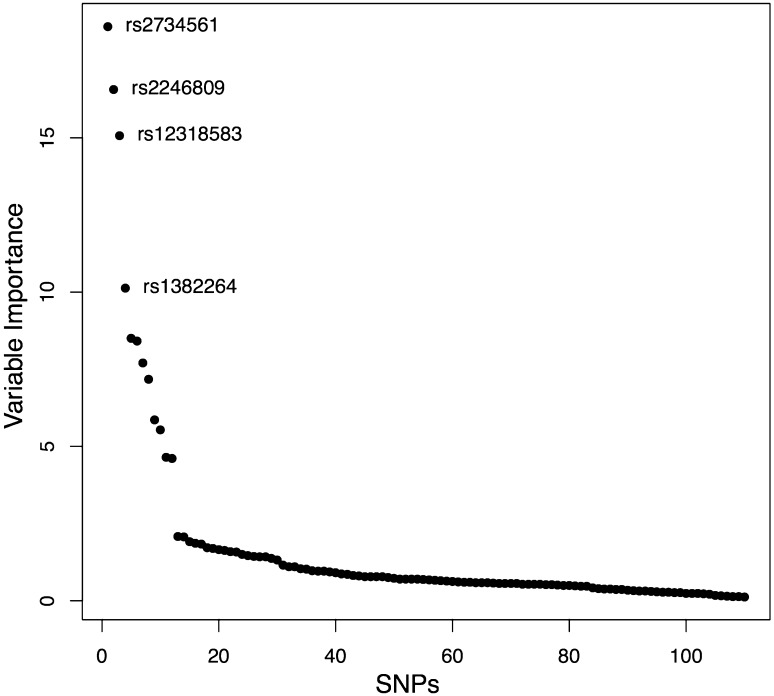
Imputation of *KLRC2* from SNPs. Variable importance in random forest classification. A small number of SNPs had importance for a predictive model. The model was dominated by the effects of three SNPs of large importance to the decision forest. The three most important SNPs were rs12318583 (*y* = 17.0352561), rs2734561 (*y* = 16.1572070) and rs2246809 (*y* = 12.9735736)

A new method for imputation of NKG2C/*KLRC2* genotypes from SNP genotyping data was developed. *KLRC2* deletion variant genotypes were imputed from commercial SNP genotyping array results in 1090 TS cases and 1531 controls from the Gambia. One hundred and eleven SNPs were found to be in linkage disequilibrium (LD) with the *KLRC2* deletion variant (*R*^2^ > 0.2). The SNP in strongest LD with the variant was rs12318583 (*R*^2^ = 0.937), followed by rs2246809 (*R*^2^ = 0.889) and rs2734561 (*R*^2^ = 0.888). Figure 1 shows the genomic region of the *KLRC2* deletion variant and highlights SNPs in the region that are in LD with this variant. Figure 2 shows the relative variable importance of each of 111 SNPs in Random Forest classification. The 111 SNP Random Forest was applied to the 309 specimens of the training set and imputation was found to be accurate in 291/309 (94.17 %) of specimens. Table 3 shows concordant and discrepant calls between the genotyped and imputed data. The three SNPs in highest LD with the *KLRC2* deletion were found to be the components of the best classifier model, which correctly imputed the genotype in 292/309 (94.5 %) specimens (Table 4).

**Table 3:** Random forest agreement with experimental data in training set, 111 SNP model

Imputed genotype	Experimental genotype		
	wt/wt	wt/del	del/del
wt/wt	119	2	0
wt/del	10	127	3
del/del	0	3	45

Cohen’s Kappa = 0.91 unweighted (CI 0.86–0.95), weighted 0.94 (CI 0.91–0.97)

**Table 4:** Selection of best classifier model (SNPs 1-5)

Model	Accuracy *n*/309 (%)	Cohen’s Kappa, unweighted	Cohen’s Kappa, weighted
rs2246809	289 (93.5)	0.90 (0.85–0.94)	0.94 (0.91–0.96)
rs2246809 + rs2734561	289 (93.5)	0.90 (0.85–0.94)	0.94 (0.91–0.96)
rs2246809 + rs2734561 + rs12318583	292 (94.5)	0.91 (0.87–0.95)	0.95 (0.92–0.97)
rs2246809 + rs2734561 + rs12318583 + rs1382264	291 (94.1)	0.91 (0.86–0.95)	0.94 (0.91–0.97)
rs2246809 + rs2734561 + rs12318583 + rs1382264 + rs1382264	292 (94.5)	0.91 (0.87–0.95)	0.95 (0.92–0.97)

The three SNP Random Forest was applied to 1010 Gambian cases of TS and 1419 controls from the same population. The imputed genotype counts were 932 (wt/wt), 1138 (wt/del) and 359 (del/del). The imputed *KLRC2* genotypes were in Hardy–Weinberg equilibrium (*p* = 0.74). There was no statistically significant association between the *KLRC2* genotype and TS in this population (Table 5). These results are consistent with our results in Gambians obtained by direct SSP-PCR genotyping (Table 2). The greater sample size (*N* = 2621) provided increased power (85 %) to detect smaller effect sizes (1.2) at the 0.05 significance level. Altogether, the results suggest that there is no association between *KLRC2* genotype and trachomatous disease, which imparts an increase in relative risk of disease that is greater than 1.2.

**Table 5:** Association between TS and *KLRC2* genotype in 2429 Gambian specimens

	West-Africans (*N* = 2429)		
	OR (95 % CI)	*Z*	*p* value
Genotype			
wt/wt	1	–	–
wt/del	1.093 (0.998–1.196)	0.980	0.327
del/del	1.138 (1.002–1.291)	1.018	0.309
Gender	1.389 (1.272–1.517)	3.732	0.0002
Age	1.007 (1.004–1.008)	3.420	0.0006

### *KLRC2* deletion is present at higher frequencies in West-African populations

The frequency of *KLRC2* deletion allele was compared among different populations (Table 6). Previous studies examining the frequency of *KLRC2* deletion have reported frequencies ranging within 17–23 % in different European and East-Asian populations (Miyashita et al. 2004; Moraru et al. 2012b; Rangel-Ramirez et al. 2014; Thomas et al. 2012). Li et al. have compiled this information and demonstrated that, with the exception of the Mexican mestizo population where the frequency is lower (10.3 %), the frequency of *KLRC2* deletion among different European and East-Asian populations is in general comparable (Li et al. 2015; Rangel-Ramirez et al. 2014). We investigated the frequency of *KLRC2* deletion among different African populations. In East-African Maasai Tanzanians, the frequency of *KLRC2* deletion (20.9 %) was comparable to the frequencies observed in Europeans (*p* = 0.398) and East-Asians (*p* = 0.773). In contrast, we found strong evidence (*p* = 1.110 × 10^−6^) that the *KLRC2* deletion allele is present at higher frequency in West-African populations (33.2 %) (Table 6). The frequency of the *KLRC2* deletion allele was the highest among Gambians (36.2 %; *p* = 2.105 × 10^−8^) followed by Bissau-Guineans (26.8 %; *p* = 0.050). West-African populations have approximately two times the odds of having the *KLRC2* deletion allele when compared to East-African Maasai Tanzanians (OR = 1.88; 95 % CI 1.46–2.43). Conversely, the odds of having the *KLRC2* deletion are about two times lower (OR = 0.44; 95 % CI 0.31–0.61) among Mexican mestizos (10.3 %) compared to Maasai Tanzanians. Collectively, the results suggest that *KLRC2* deletion is found at different levels within distinct human populations.

**Table 6:**
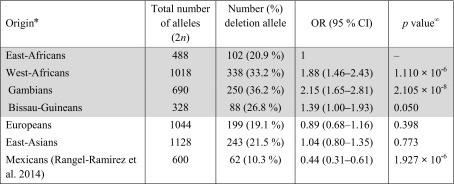
Odds ratios for the association between the frequency of the *KLRC2* deletion allele and origin of the study population

* East-Africans (Tanzanians Maasai); West-Africans (Gambians, Bissau-Guineans); Europeans [Germans (Thomas et al. 2012), Dutch (Miyashita et al. 2004) and Spanish (Moraru et al. 2012b)]; East-Asians [Chinese (Li et al. 2015) and Japanese (Miyashita et al. 2004)]; grey shade = this study (all individuals without TS included); genotypes in Hardy–Weinberg equilibrium

*OR* odds ratio, *CI* confidence interval

^∞^Wald-test

## Discussion

Deletion of *KLRC2* gene was first reported in 2003 in a Japanese population (Hikami et al. 2003) and subsequently in other contemporary human populations (Li et al. 2015; Miyashita et al. 2004; Moraru et al. 2012b; Rangel-Ramirez et al. 2014; Thomas et al. 2012). Goodier et al. measured the frequency of the deletion haplotype in a small cohort of Gambians. Consistent with our results, the frequency of the deletion haplotype in this cohort was higher than documented elsewhere (Goodier et al. 2014). Our study is the first to report the frequency of *KLRC2* deletion in different African populations. The deletion haplotype is present at relatively high frequencies in distinct human populations ranging from approximately 10 % in Mexican mestizos (Rangel-Ramirez et al. 2014) to 36 % in West-African Gambians.

We have confirmed that copy number variation at the *KLRC2* locus correlates with the expression levels of functional NKG2C receptor on NK cells as previously reported (Muntasell et al. 2013; Noyola et al. 2012; Thomas et al. 2012). The high population frequency of *KLRC2* deletion that disrupts the expression of the receptor suggests that NKG2C is not essential for survival and other receptors might compensate for the lack of NKG2C on immune cells. CMV expanded NKG2C positive cells also express inhibitory KIR receptors specific for self–HLA-C (Beziat et al. 2012, 2013; Djaoud et al. 2013; Foley et al. 2012b; Horowitz et al. 2013). It is conceivable to hypothesise that KIR or other receptors can compensate for the lack of NKG2C in *KLRC2* negative individuals so that NKG2C deletion can be maintained without higher costs in populations. Future studies looking at the function and receptor diversity of CMV expanded NK cells in NKG2C positive and NKG2C negative individuals are likely to address some of these questions.

Myashita et al. hypothesised that the *KLRC2* deletion has emerged as a result of an unequal ancient crossover event. Shared nucleotides exclusive to the deletion haplotype in individuals from distinct populations (Miyashita et al. 2004) along with the global distribution of the deletion haplotype suggest that the deletion originated from a common ancestral event. The exact origin of the deletion event and what form of selection (for instance balancing or frequency dependent selection) drove the maintenance of the deletion haplotype at different frequencies in the different populations are currently not understood.

Of particular notice is the lower frequency of the KLRC2 deletion in Mexican mestizos (10.3 %; *p* = 1.927 × 10^−6^) and the higher frequency of the *KLRC2* deletion in West-African populations from The Gambia and Guinea-Bissau (33.2 %, *p* = 1.110 × 10^−6^). Interestingly, HLA-C2 epitopes as well as centromeric KIR B haplotypes have also been found at higher frequencies in West Africans (Hollenbach et al. 2012; Roberts et al. 2014). Differential selective pressures, patterns of modern human migrations, admixture, bottlenecks and gene flow may all account for these observations. The results are consistent with evidence that supports an African origin of anatomically modern humans who have consequently migrated into Eurasia and later spread into other parts of the globe including the Americas (Campbell and Tishkoff 2008), where a high level of genetic drift is expected. Rangel-Ramirez et al. believe that the frequency of the *KLRC2* deletion might be even lower in Native American populations as compared to the mixed Mexican mestizo population, which also has Caucasian origin (Rangel-Ramirez et al. 2014). Our data are further supported by research on African genetic structure where a differential gene clustering has been observed between East- and West-African populations (Tishkoff et al. 2009). Due to the variable degrees of population movements, expansions and admixture between Africans, different modern and ancient hunter-gatherer populations, West Eurasians and even archaic introgression (Gallego Llorente et al. 2015; Gurdasani et al. 2015; Racimo et al. 2015), one cannot exclude a more recent origin of the *KLRC2* deletion. Llorente et al. have recently shown that the Eurasian component in African populations is higher than previously expected with the highest levels of admixture found in East Africa but extending all the way to West and Southern Africa, with some African populations showing up to 7 % of their genomes to be of Eurasian origin (Gallego Llorente et al. 2015).

As humans migrated and settled into different environments, they were exposed to a variety of pathogens and other environmental pressures. Natural selection of alleles that confer advantage to new ecosystems occurs for both humans and pathogens. Infectious diseases are among the most predominant selective pressures that act on humans throughout evolution (Fumagalli et al. 2011). Since the *KLRC2* deletion is present in different populations, we hypothesise that it may provide selective advantage to a particular environment. Since both wild-type and deletion alleles can be found at high frequencies in the population, a balancing selection that favours diversity seems to be the most plausible model. In fact, a wide variety of immune response genes including the major histocompatibility complex (MHC) and KIR haplotypes are thought to be maintained in human populations by balancing selection (Karlsson et al. 2014; Parham and Moffett 2013). This mechanism favours diversity, which is required to deal with the broad range of pathogens that affects humans. In the case of the KIR haplotypes it has been proposed that the balanced selection of KIR A and B haplotypes in all human populations is related to the distinct and complementary functions of NK cells in both immunity and reproduction (Parham and Moffett 2013). The role of *KLRC2* deletion in reproduction has not been addressed so far and it would be interesting to study whether *KLRC2* deletion has any impact in this context. In the context of infection, *KLRC2* deletion was shown to be associated with impaired outcomes to CMV, HIV infection and psoriasis (Goodier et al. 2014; Thomas et al. 2012; Zeng et al. 2013). In this study, we have found no evidence for an association between *KLRC2* deletion and trachoma in different African populations where trachoma has historically been hyper-endemic. It is, therefore, unlikely that any of the diseases studied to date are responsible for the maintenance of the *KLRC2* deletion in human populations. Even though we were well-powered (85 %) to detect small effect sizes (as low as 1.2) at the 0.05 significance level, we cannot rule out that common sources of bias (such as the non-differential number of samples excluded from the study due to genotyping failure or residual confounding) may have shifted the association towards the null. We have addressed potential sources of bias by validating and optimising our genotyping method against flow cytometry and a panel of reference cell lines and we have obtained a low genotype failure rate of 2.5 %. We have also confirmed that TS is associated with age and gender as previously described (Courtright and West 2004; Wolle et al. 2009). In these analyses, we did not make corrections for pairwise kinship between individuals. We have previously identified (Roberts et al. 2015) that cryptic kinship exists in the Gambian population in which we are working and this was demonstrated to cause genome-wide inflation of association test statistics in a genome-wide association scan. In order to control for cryptic kinship in these analyses, we would have needed access to high-density SNP genotyping for all participants and in all three populations. Such data were not available and in the context of a null result, we expect that inflation of the test statistic was not a problem that needed to be addressed here.

Contrary to KLRC2 deletion, HLA-C2 and KIR2DL2/2DL3 heterozygosity was shown to be associated with conjunctival scarring (Roberts et al. 2014) and a number of different KIR and HLA constellations have been shown to be associated with various infectious diseases, reproduction and survival. Altogether, these results suggest that NK cell receptors are predominant targets of selective pressures and that these pressures come from highly prevalent infectious diseases, highly selective prenatal and perinatal complications; as well as the double-edged threat of infection related infertility, for example due to genital *C. trachomatis* infection.

Further genetic association studies in additional human populations would be required to understand how and why the deletion has been maintained in humans. It would also be of interest to investigate why the deletion is present at such a high frequency in West Africa, and the role of NKG2C in pregnancy might warrant further research.

In order to investigate the impact of large genetic deletions with a high degree of homology with other genes in the same genomic region (such as *KLRC2* and other *KLRC* genes in the NKC region), a candidate gene approach is often more appropriate than genome wide association studies (GWAS). On the other hand, GWAS provide large and well-documented cohorts of patients that offer more power to detect disease associations and signatures of selection. GWAS can be further exploited for genetic analysis such as copy number variations (Hinds et al. 2006; McCarroll 2008). We have shown that information contained in three SNPs is enough to predict *KLRC2* genotypes with an accuracy of 94.5 %. Even though the results need to be validated in other populations, this method could in principle be used to impute *KLRC2* genotype data from a variety of different populations for which SNP genotyping array data are available. Taken alone, the SNP rs2246809 is also highly accurate as a classifier tag-SNP, meaning that a simple SNP genotyping assay could replace the cumbersome agarose PCR approach and achieve more than 90 % accuracy in classification of samples which have not previously been genotyped by any method. This would provide an easier and faster alternative to advance our knowledge on the relevance of *KLRC2* deletions in human populations. The method described to identify SNPs in LD that can be used in genotype classification can be easily applied to other gene deletions or regions where GWAS data are available in large bio-bank collections. The three SNPs used in the model were all imputed SNPs, rather than having been directly genotyped in the GWAS panel. This means that there is no additional burden in using three as opposed to one SNP, whilst there is some marginal gain in using the full model.

Genotyping complex loci such as KLRC2, KIR and HLA is both methodologically challenging and expensive. The development of simple and user friendly imputation tools such as this Random Forest implementation for KLRC2 immunogenetic typing presents opportunities for many research groups, without resources to conduct GWAS, to access large publicly available data-sets. This is complementary to recent papers that have described tools for HLA (Zheng et al. 2014) and KIR (Vukcevic et al. 2015) imputation, some of which (Levin et al. 2014) have been applied in specimens from people of African descent. As we perform more immuno-genotyping in cohorts that have also been tested using high-density SNP genotyping arrays, we approach a tipping point in immuno-genomics, where methodologically complex genotyping methods can begin to be replaced with statistical models of prediction that utilise more widely available SNP data.

## Electronic supplementary material

Below is the link to the electronic supplementary material.

Supplementary material 1 (DOCX 765 kb)

Supplementary material 2 (DOCX 26 kb)

Supplementary material 3 (DOCX 25 kb)

## Abbreviations

NK: Natural killer
*KLRC2*: Killer cell lectin-like receptor C2
TS: Trachomatous scarring
TF: Trachomatous inflammation-follicular
