# Supplementary material for: Differential frequency of NKG2C/KLRC2 deletion in distinct African populations and susceptibility to Trachoma: a new method for imputation of KLRC2 genotypes from SNP genotyping data
Source: Hum Genet. 2016 Jun 16;135:939–51. doi: 10.1007/s00439-016-1694-2 (PMC4947484; doi:10.1007/s00439-016-1694-2)
Supplement: Supplementary file 1 — Supplementary material 1 (DOCX 765 kb) [file 439_2016_1694_MOESM1_ESM.docx]

**A B**

NKG2C MFI

******C**

CD56

CD3

**Supplementary Figure 1. Validation of the NKG2C/*KLRC2* genotyping method and correlation with flow cytometry expression data.** Deletion and wild-type alleles can be detected by SSP-PCR with two specific primer pairs that yield a 200bp and a 411bp product for the detection of wild-type and deletion alleles, respectively. Primer pairs and genotyping method were validated against a panel of published (Moraru et al. 2012) reference cell lines (A). *KLRC2* genotypes from a sample of the study population (N=76) correlate with NKG2C protein expression at the surface of CD3^-^CD56^+^ NK cells (B,C) as previously reported (Muntasell et al. 2013; Noyola et al. 2012; Thomas et al. 2012).
