# Supplementary material for: Differential frequency of NKG2C/KLRC2 deletion in distinct African populations and susceptibility to Trachoma: a new method for imputation of KLRC2 genotypes from SNP genotyping data
Source: Hum Genet. 2016 Jun 16;135:939–51. doi: 10.1007/s00439-016-1694-2 (PMC4947484; doi:10.1007/s00439-016-1694-2)
Supplement: Supplementary file 2 — Supplementary material 2 (DOCX 26 kb) [file 439_2016_1694_MOESM2_ESM.docx]

**Supplementary Table 1. Adjusted odds ratios (age, gender and ethnicity) for the association between *KLRC2* genotypes and Trachomatous Scarring (TS); estimated by logistic regression.**

|  | West-Africans (N=787) | | | |
| --- | --- | --- | --- | --- |
|  | **OR** | **95% CI** | **p-value*** | |
| wt/wt | 1 | --- | --- | 0.852 |
| Genotype wt/del | 1.03 | 0.75-1.40 | 0.873 |  |
| del/del | 1.14 | 0.72-1.81 | 0.574 |  |
| Age | 1.02 | 1.01-1.02 | **3.512 x 10^-6^** |  |
| Gender | 0.80 | 0.58-1.09 | 0.159 |  |
| Mandinka | 1 | --- | --- |  |
| Ethnicity Jola | 0.77 | 0.54-1.11 | 0.160 | 0.239 |
| Others | 1.03 | 0.72-1.49 | 0.842 |  |

West-Africans (Gambians: 39.9% Mandinka; 30.0% Jola and 30.1% from other ethnicities); OR=Odds ratio (adjusted for age, gender and ethnicity); CI=Confidence Interval; *Wald test (left), Likelihood Ratio Test (right)
