# Supplementary material for: Differential frequency of NKG2C/KLRC2 deletion in distinct African populations and susceptibility to Trachoma: a new method for imputation of KLRC2 genotypes from SNP genotyping data
Source: Hum Genet. 2016 Jun 16;135:939–51. doi: 10.1007/s00439-016-1694-2 (PMC4947484; doi:10.1007/s00439-016-1694-2)
Supplement: Supplementary file 3 — Supplementary material 3 (DOCX 25 kb) [file 439_2016_1694_MOESM3_ESM.docx]

**Supplementary Table 2. Adjusted odds ratios (age and gender) for the association between *KLRC2* genotypes and Trachomatous Inflammation-Follicular (TF) in children; estimated by logistic regression.**

|  | West-African Children (N=226) | | | |
| --- | --- | --- | --- | --- |
| Genotype | **OR** | **95% CI** | **p-value*** | |
| wt/wt | 1 | --- | --- | 0.617 |
| wt/del | 0.77 | 0.44-1.32 | 0.338 |  |
| del/del | 0.78 | 0.27-2.32 | 0.660 |  |
| Age | 0.98 | 0.88-1.08 | 0.649 | 0.649 |
| Gender | 1.01 | 0.60-1.72 | 0.960 | 0.960 |

West-African children (Gambians N=62, Bissau-Guineans N=164); OR=Odds ratio; CI=Confidence Interval; *Wald test (left), Likelihood Ratio Test (right).
